# Game-based digital therapeutics for children and adolescents: Their therapeutic effects on mental health problems, the sustainability of the therapeutic effects and the transfer of cognitive functions

**DOI:** 10.3389/fpsyt.2022.986687

**Published:** 2022-11-29

**Authors:** Eunhye Choi, Eun-Ha Yoon, Min-Hyeon Park

**Affiliations:** Department of Psychiatry, Eunpyeong St. Mary's Hospital, Catholic University of Korea, Seoul, South Korea

**Keywords:** children and adolescents, mental health problem, digital therapeutics, therapeutic effects, transfer, sustainability, video games

## Abstract

Mental health problems in childhood and adolescence (e. g., attention deficits, problems in emotional understanding and subclinical levels of anxiety) are reported to develop further in adulthood and/or to increase the risk of developing mental health disorders in adulthood. Although it is important to provide treatment or prevention interventions for children and adolescents in order to reduce the risk of the further development of mental health problems, the pharmacological and behavioral treatments for this age group have limitations (e.g., limited access, unsustainable treatment effects and the lack of engagement in intervention). In order to overcome the limitations of conventional treatments, the use of digital technology, especially video games for this age group, is suggested. In order to be accepted as clinical interventions, objective evidence for the therapeutic effects of digital therapeutic that used video games to treat or prevent targeted mental health problems are required. Thus, this review aims to explore whether game-based digital therapeutics (DTx) for children and adolescents showed therapeutic effects on targeted mental health problems. As game-based DTx are suggested to show sustained therapeutic effects and the transfer of cognitive functions, it also reviews the maintenance of the therapeutic effects of DTx and the extent of the transfer of cognitive functions. Game-based DTx that are developed to treat or prevent mental health problems (e.g., attention deficit, depression) in children and adolescents are found to show therapeutic effects on targeted mental health problems despite the limitations (e.g., small sample size, limited investigation of the sustainability). This review would contribute to the understanding of whether there is objective evidence of the therapeutic effects of digital therapeutics using video games that deliver treatment or prevention interventions for mental health problems in children and adolescents.

## Introduction

More than 10% of children and adolescents in the world are reported to have mental health problems (1). With the improvement in diagnosis systems (2), there is an increase in the clinical diagnosis of neurodevelopmental disorders (e.g., attention deficits) in this age group compared to the past (3). An increased prevalence of affective disorders (e.g., depressive symptoms) in childhood and adolescence is also reported (3). Clinical or subclinical mental health problems (e.g., attention deficit, anxiety and depressive symptoms) in childhood and adolescence appear to be persistent into adulthood (4). About 50% of young adults show mental health disorders that are continued from their childhood psychiatric disorders (5). Moreover, children and adolescents, who report clinical or subclinical mental health conditions, are more likely to develop mental health problems later in their adulthood (6–8). As mental health problems in children and adolescents tend to be persistent and recurrent in their adulthood (9), it is important to provide effective interventions for mental health problems in this age group (10, 11).

However, a lot of children and adolescents are estimated to have difficulties receiving the interventions for their mental health problems (12) due to two barriers. One barrier is the side effects of pharmacological treatments that limit treatment options for this age group (13, 14). Pharmacological treatments that show side effects (e.g., headaches and insomnia) in some patients with attention deficits (13) are not acceptable for some children with attention deficits (15). In case of antidepressants, their safety and effectiveness in adolescents have been argued (16) despite the increased use of them in this age group (17). The other barrier is the limited access to the treatments (18). The number of adequately trained therapists or the availability of facilities is not sufficient to meet the needs for overall mental health problems (11, 19). In case of neurofeedback that aims to treat attention deficit hyperactivity disorder (ADHD), it offers visual or auditory reinforcement in order to alter activities of relevant brain regions (20, 21) that are found to show specific electroencephalography (EEG) patterns in children with ADHD (22). That is, as its treatment effects on ADHD is highly dependent on the ability of clinicians to establish links between the symptoms of a patient and his or her dysregulated EEG patterns in brain regions (20), the accessibility to the clinicians with the adequate level of trainings is required. Furthermore, the COVID-19 that results in social distancing and lockdown in some countries makes the access to face-to-face interventions even more limited (23). In order to successfully offer the interventions for mental health problems in children and adolescents by overcoming the barriers, it has become important to develop more innovative and cost-effective intervention methods for this age group (24).

Digital technology is suggested as a delivery tool for automated and self-directed interventions by improving the accessibility (25). Software-driven therapeutic interventions that aim to prevent, manage, or treat medical or chronic diseases are referred to as Digital Therapeutics (DTx) (26). Unlike wellness or other digital health products that deliver more personalized clinical care based on the understanding of health-related behaviors through digital technology (27, 28), DTx are developed to target a specific disorder or disease (29). DTx could target the conditions that are difficult to be intervened in frequently used interventions (30). As DTx are regarded as “an emerging class of medicines” (31), they also have obtained the approval of the relevant regulatory authorities (e.g., the U.S. Food and Drug Administration) (30) based on clinical evidence for the effectiveness similar to conventional medicines and medicine supplies (29). DTx that could decrease time demands of clinicians during the interventions (26) are found to be effective in the improvement of various clinical conditions (e.g., the impairment of sensorimotor functions, cognitive deficits and depression) by replacing or complementing other intervention methods (32). Among various technological platforms and systems that have been facilitated to enhance the delivery of healthcare services (26), video games (VGs) that can be helpful for mental health problems (e.g., emotion regulation, stress management, attention deficits and depression) are suggested as an attractive and immersive delivery tool for clinical interventions in children and adolescents (33, 34).

VGs, one of the most entertaining activities for children and adolescents (35), could provide them with more flexible and personalized learning environments (36) by maintaining the optimal level of challenges for players in response to their actions (37). Engagement issues and the limited consideration of individual differences in learning process are reported to limit the effective delivery of mental health interventions. For example, people with ADHD have difficulty managing their brain activities repeatedly over neurofeedback training sessions despite its effectiveness (20). The motivation level and the extent of engagement that children and adolescents show in cognitive trainings [e.g., Rational Emotive Behavior Education (REBE) interventions that focused on the cultivation of rational beliefs (11), cognitive behavioral therapy (CBT)] are also not stable during the intervention process (38, 39). That is, despite their effectiveness for anxiety symptoms (39), the way to deliver the interventions seems to be related to the decreased effectiveness (35, 40). Moreover, some children and adolescents (e.g., those with attention deficits) show difficulties learning new abilities in CBT that focuses on the shift of the anxious state trough relaxation trainings (37) and need more time to learn these skills (41). It is found to be difficult to consider the diversity of learning needs and individual differences in learning paces of children in the interventions (37).

Furthermore, the sustainability and generalization of therapeutic effects outside the intervention periods [e.g., (42)] are concerns that should be considered in order to improve the effectiveness of the interventions for mental health problems. Pharmacological and behavioral treatments show unsustainable therapeutic effects over time (43, 44). Practice activities for the generalization of skills in CBT (e.g., role-playing and homework assignments) are not effective for various reasons (e.g., limited time and subjective boredom) (37) and learned skills in traditional biofeedback interventions show limited generalization in real-world settings despite their effectiveness on the reinforcement of emotional relaxation abilities (45). However, VGs could offer opportunities for the generalized practice of learned skills by making players feel a strong sense of agency, entertainment and rewards (37). Thus, implementing VGs or gaming elements (e.g., meaningful contexts and scenarios to serve learning objectives) (41, 46) in the interventions for mental health problems of children and adolescents is suggested to have the potential to overcome the limitations of frequently used mental health interventions.

Taken together, despite the increasing needs for mental health interventions in children and adolescents, intervention options for this age group are limited and effective interventions report concerns that could influence the effectiveness during and after the interventions. The implementation of digital technology is suggested as alternative treatment or prevention interventions for mental health problems (23). Among various options of digital technology, VGs are found to be cost-effective (37) and age-appropriate (33, 34) delivery tool for children and adolescents with mental health problems. Although DTx using VGs are suggested to have the potential to treat or prevent mental health problems in children and adolescents by overcoming concerned issues of frequently used interventions, the objective evidence for the effectiveness of DTx is not sufficient (47). The safety and efficacy of DTx should be proved for the acceptance for DTx by patients and clinicians (48), and some game-based DTx do not show sustained interventions effects (32) despite the potential for the generalization of skills that are learned in interventions (37, 41). Thus, this study aims to review whether game-based DTx show therapeutic or preventive effects on targeted mental health problems in children and adolescents. It also reviews the sustainability of the therapeutic effects of game-based DTx. Moreover, the extent of transfer of cognitive functions in game-based DTx for children and adolescents is reviewed. Not only intervention outcomes (e.g., decreased risk of falls) but also cognitive functions (e.g., processing speed and selective attention) were improved in older individuals (e.g., those who aged more than 60 years) who received game-based trainings (49–52). Improved cognitive functions after the game-based training (i.e., *NeuroRacer*) were comparable to cognitive functions of 20-year-olds and were maintained for 6 months along with alterations in brain activation (53). The results of studies in the non-clinical population suggested that implementing VGs has the potential for neuroplasticity. However, playing VGs are found to be related to near transfer of cognitive functions [i.e., the improvement of cognitive functions (e.g., attention) that are related to characteristics of VGs] (54, 55). That is, the VG-related neuroplasticity is limited to the brain regions that are related to characteristics of VGs. As the VG-related neuroplasticity could influence the therapeutic effects of DTx, the transfer of cognitive functions in children and adolescents with subclinical or clinical levels of mental health problems after game-based DTx interventions is additionally reviewed.

## Methods

Literatures search was conducted in databases (e.g., PubMed and Web of Science) and Google Scholar with search terms (e.g., “digital therapeutics”, “video game digital therapeutics”, “digital therapeutics for children and adolescents”, and “game-based digital therapeutics for children and adolescents”) without the restriction of the publication date. In the process of screening searched literatures, not only the age of children and adolescents but also the measurements for therapeutic effects of DTx were not limited. Although DTx usually get approval from the regulatory institutions (30), game-based DTx for mental health problems in children and adolescents are an emerging research field where only one game-based DTx (i.e., EndeavorRx) for this age group was identified to be FDA-approved (56). Thus, game-based DTx in this study is defined as interventions that use VGs as a delivery tool to treat or prevent targeted mental health problems in children and adolescents with or without the approval from regulatory authorities. Mental health problems in the review encompass both neurodevelopmental and affective disorders that show an increasing trend in childhood and adolescence (3). Through the process of screening the searched literatures, seven game-based DTx for this age group were identified. In order to explore all relevant literatures that examined therapeutic or preventive effects of identified DTx, the additional search of literatures was conducted by using names of DTx that were indicated in literatures (e.g., “Project:EVO” and “RAGE-Control”) as additional search terms. After the additional search, abstracts were screened to identify whether children and/or adolescents were included as participants and whether the therapeutic effects of identified DTx were examined. The inclusion criteria of the literatures in this review were (1) full-text original research articles published in English and (2) the examination of therapeutic or preventive effects of game-based DTx that aimed to target mental health problems in children and adolescents. That is, DTx that are not developed to target mental health problems in children and adolescents and that do not include gaming elements as a delivery tool were not included. Research articles that did not investigate therapeutic or preventive effects of identified DTx in children and/or adolescents were also excluded. As a result, 22 research articles that met the inclusion criteria were included in this review.

## Therapeutic effects of DTx for children and adolescents

Using digital devices is suggested to have the potential for the rehabilitation of cognitive functions by enhancing the neuroplasticity (32). Based on the potential effectiveness of game-based DTx for the cognitive rehabilitation (32), there are game-based DTx that are developed for children with ADHD or autism spectrum disorder (ASD) who show impairments in attention function and inhibitory control (44, 57). Children and adolescents are reported to have not only neurodevelopmental disorders but also affective disorders (3). Some children and adolescents report difficulties regarding emotional understanding that is related to the further development of emotional disorders (11). Some of them report subclinical levels of anxiety or depression (37, 58). That is, there are also game-based DTx that aim to treat or prevent affective disorders in children and adolescents. That is, seven game-based DTx that were identified through the literature search in databases are found to target attention deficits, emotional regulation, anxiety or depressive symptoms in this age group (see Table 1). While REThink aims to offer prevention intervention by making children and adolescents learn emotion regulation skills (59), other six game-based DTx aim to offer treatment interventions for targeted mental health problems.

**Table 1:** The explanation of seven DTx for children and adolescents.

**Name of DTx**	**The focus of the intervention**	**Explanation**
EndeavorRx	attention	EndeavorRx, which is referred as “Project: EVO” or “AKL-T01” in literatures, is developed by Akili Interactive based on the *Neuroracer* model (42, 60). In order to treat children with attentional deficits (age range = 8–12 years), it uses gaming elements (e.g., action VG graphics, and real-time adaptive mechanisms) (42, 44, 61). It is approved by the FDA as the first DTx that delivered the treatment by using VG and is prescribed for children with ADHD (56). The children are asked to play it for 25 minutes daily on five days of a week during the prescribed period (i.e., 4 weeks) (44, 61).
ATENTIVmynd	attention	ATENTIVmynd, which is referred as ‘Brain-computer Interface' in literatures, is developed by Neurosky Inc (4). It deploys an EEG-headband that is used to calibrate the optimal state of attention and to track attention states during the game play (20) with the training game, CogoLand, where children navigate the environment by controlling an avatar (4). The speed of the avatar's movement is modulated by the level of their concentration (4). The training consists of about 24 sessions between 6 and 8 weeks and each game takes about 20–25 min for children to play through an end (62).
RECOGNeyes	attention	RECOGNeyes is developed by University of Nottingham. Players learn the way to control different aspects of visual attention by using their eyes as a game controller (63).
REThink	emotion regulation	REThink is a standalone application that is adapted to be used in tablets as a prevention tool in children and adolescents from the general population (59). It helps them to build psychological resilience by including main character, RETMAN, who supports the learning of healthy strategies to cope with dysfunctional negative emotions (e.g., anxiety and anger) (11, 59). Based on the principles of REBT and REBE interventions, REThink consists of seven levels with specific objectives (e.g., “identifying emotional reactions”, “identifying cognitive processes”, and “building relaxation skills”) (11).
Mightier	emotion regulation	Mightier, which is referred as RAGE Control in literatures, is developed in Boston Children's hospital and Harvard Medical School (64). In order to improve emotion regulation skills in children and adolescents, it uses biofeedback and the relaxation in the modified VG design inspired by a classic VG, Space invader (45, 65, 66). That is, its storyline is to defend the attack of aliens as a hero by controlling a space ship and firing spaceships of aliens (65). During the game playing, their emotional arousal (i.e., heart rate) is taken as an input (45) that moderates the activity of firing a bullet (65). With the progress in the game, “friendly spaceships”, which is an additional challenge for inhibitory control, are introduced (65).
MindLight	anxiety	It is developed by Play Nice Institute and Gain Play studio (67). It deploys three evidence-based strategies for the reduction of anxiety in children and adolescents (age range: 8–16 years): (1) neurofeedback training (i.e., the guidance to keep their EEG waves consistent throughout the play), (2) exposure training (i.e., a validated CBT treatment component for children to practice various relaxation techniques) and (3) attention bias modification (i.e., modified attentional system to attend more to positive stimuli) (37). Its storyline is to save a grandmother from evil forces by exploring her dark mansion, chasing away or uncovering “fear events” and solving problems (68). In the exploration process that provokes the anxiety, children use their MindLight of which brightness is associated with the real-time relaxation state measured by the EEG headset (68).
SPARX	depression	SPARX is an interactive fantasy game that is developed for adolescents with depression (8, 58). The adolescents choose, customize and control their avatars in order to go through seven challenges, related to CBT components (e.g., relaxation training, social skills and cognitive restructuring) (69), for the completion of the mission (i.e., the removal of the world of gloom and negativity) (58, 70).

REBT stands for Rational Emotive behavioral Therapy, RAGE Control stands for Regulate And Gain Emotional Control, SPARX stands for Smart, Positive, Active, Realistic, X-Factor Thoughts.

### EndeavorRx

Children with ADHD showed a high level of adherence when playing EndeavorRx (42, 44, 57). EndeavorRx that was feasible in home environments and acceptable in the children (42, 57, 60) was found to significantly improve attention function in not only children with attention deficits (44, 71) but also those without the deficits (42, 60). Children, who had ASD and ADHD, also showed a trend of the improved attention function and inhibitory control (57). That is, based on parental reports, the effectiveness of EndeavorRx on decreased ADHD symptoms was found in children who had ASD and ADHD (57), children who had a recent history of pharmacological interventions for ADHD (44), and children who had ADHD without other comorbidities (71). The intervention-related improvement in attention functioning was found to be affected not by the changes in basic motoric speed but by the intervention (60, 71). Moreover, the extent of improvement in attention function through the EndeavorRx intervention was greater in children with a higher level of attention deficits (42, 60) and the improvement of attention resulted from the movement of more children, who received the intervention, into the normative ranges of attention function (44). Both one third of children who had sensory processing dysfunction (SPD) and inattentive-ADHD (60) and 44% of children who had ADHD without other comorbidities (71) did not meet the clinical threshold for inattention after the intervention. Their performance regarding attention after the intervention was comparable or even better than the baseline attention performance of typically developing children (60). Furthermore, as parental reports of reduced inattentive behaviors were associated with significantly increased middle frontal theta (MFT) activity power (60), playing EndeavorRx was related to the changes in the activations of brain regions for attention function (60, 71). Unlike the significant increases in MFT in the middle of the attention task in children with SPD and inattentive-ADHD (60), the significant increases in MFT in children who had ADHD without other comorbidities were found in the earlier and later stages of the task (71). These results suggest the therapeutic effects of EndeavorRx on attention deficits in children.

### ATENTIVmynd

With a high level of compliance with ATENTIVmynd in children (4), children with ADHD showed significant decreases in inattentive symptoms after playing ATENTIVmynd (4, 72). Both parents and clinicians reported the improvement in ADHD symptoms in children through ATENTIVmynd compared to non–pharmaceutical interventions (20). Parental reports also showed the significant improvement of hyperactive-impulsive symptoms in children with the combined subtype of ADHD (4). Children with more severe attention deficits showed greater effects of ATENTIVmynd intervention (4). Moreover, children with ADHD showed the reorganization of the functional networks (e.g., decreased functional connectivity within the salience network), which was associated with the improvement of inattentive symptoms and the reduction of internalizing problems (72). ATENTIVmynd showed therapeutic effects of targeted attention deficits in children.

### RECOGNeyes

Not adolescents but the majority of children, who were younger than 12 years, reported that playing RECOGNeyes was enjoyable and its levels of challenges were appropriate for them (63). While playing RECOGNeyes did not result in the change in the number of errors (i.e., commissions and omissions), the children and adolescents, who played RECOGNeyes by using their eyes as a controller, showed decreases in the level of impulsivity, made fewer fixations and fixated on the target for a longer duration than those who played RECOGNeyes by using the mouse as a controller (63). They also showed the improvement of impulsivity (63). That is, RECOGNeyes was found to improve the control of attention in children and adolescents with ADHD (63).

### REThink

Compared to children who received REBE intervention, children, who played REThink, reported a higher level of satisfaction in the middle of REThik intervention that was an important factor to booster the therapeutic effects (11). Playing REThink was found not only to reduce overall negative emotional symptoms (e.g., anger and anxiety) but also to improve the emotional awareness and emotional control (11). That is, the risk of the development of problems decreased in children, who played REThink, but not in children, who received REBE intervention, and those in the waitlist group (11). Moreover, despite the overall decreases in anxiety symptoms in all groups of children, children, who played REThink, showed more inhibited activation in the frontal brain regions of the right hemisphere that was associated with negative affects and the motivation to withdraw (24). That is, it was found that the effectiveness of REThink on emotion regulation was associated with changes in irrational beliefs (73). The reports of emotional symptoms and depressive moods decreased in children, who played REThink, compared to those who received REBE intervention (73). These results suggested that REThink was an engaging and effective prevention intervention for children to learn emotional regulation skills.

### Mightier

Mightier that was rated as a highly satisfactory, enjoyable and helpful intervention approach with a high level of adherence was found to be an acceptable intervention approach in children (66, 74). A case study that showed the feasibility of Mightier for the engagement and its effectiveness in coaching self-regulation skills suggested the potential that Mightier would have therapeutic effects in children and adolescents with anger and aggression (74). Compared to anger control therapy (ACT) that aimed to treat anger and aggression as one of CBT programs (75), Mightier that augmented ACT was found to significantly decrease symptoms of emotional arousal (e.g., anger) and to improve the maintenance of calm states in children and adolescents (66). As ACT did not show therapeutic effects on emotional arousal and the control of emotion (66), Mightier appeared to have the potential to be used as a standalone intervention for the emotion regulation. Moreover, as children whose primary diagnosis was a restrictive eating disorder showed improvement in anger after playing Mightier, Mightier appeared to have valuable therapeutic effects in children who showed some emotional problems regarding anger in addition to their primary mental health problems (66).

### MindLight

MindLight that was rated to produce a higher level of anxiety by children compared to the commercial control game was reported to be less appealing than the control game (37) but more equally appealing like CBT interventions (76). The effectiveness of MindLight on the reduction of anxiety symptoms in children with the subclinical level of anxiety was found to be comparable to that of commercial control games (e.g., “Max” where players control the avatar in puzzle platform VG) (37, 77). Although children, who played MindLight, showed the similar extent of improvement of anxiety symptoms compared to those who played control games immediately after the intervention (37), not only children with more severe level of anxiety but also children with ASD in addition to subclinical anxiety symptoms showed improved anxiety symptoms after playing MindLight (37, 77). The therapeutic effect of MindLight on anxiety symptoms in children with elevated levels of anxiety was also comparable to that of CBT (76). MindLight was found to be an effective intervention for anxiety without the further addition of CBT elements in that including more CBT-related components in MindLight for children with ASD did not have additional therapeutic effects on anxiety (78). Moreover, compared to boys, girls, who showed higher levels of anxiety symptoms at the beginning of the intervention, showed greater decreases in their anxiety symptoms after playing MindLight (76). These results suggested that MindLight that provided the engaging context for children to practice their strategies for emotion regulation (37) showed the therapeutic effect on the subclinical level of anxiety that was comparable to the game- or CBT-relevant improvements (37, 76, 77).

### SPARX

SPARX that was reported as a helpful and satisfactory intervention (8, 58) showed a high level of adherence in adolescents (58, 79) despite the relatively low adherence levels in the adolescent sample including transgender adolescents (69). The effectiveness of SPARX in the reduction of depressive symptoms was comparable to that of CBT in Dutch female adolescents with elevated depressive symptoms (8). Compared to usual treatments (e.g., counseling), SPARX was found to significantly reduce depressive symptoms in adolescents with mild to moderate depressive disorder (58) and adolescents who were excluded from mainstream education (79). The therapeutic effect of SPARX on depressive symptoms was greater in adolescents who showed more severe level of depression at the beginning of the intervention (58). However, SPARX did not show the improvement of depressive symptoms in transgender adolescents (69). Although SPARX did not effectively reduce depressive symptoms in some adolescents, it was suggested to have the therapeutic effect on depressive symptoms that was comparable to or better than the interventions for depression.

The identified seven game-based DTx are as effective as usual treatments (e.g., CBT) or show better therapeutic or preventive effects compared to some interventions (e.g., REBE interventions) (see Table 2). EndeavorRx, ATENTIVmynd and RECOGNeyes that target attention deficits show therapeutic effects in children and adolescents with ADHD (4, 44, 57, 63, 72). REThink and Mightier result in decreased symptoms of negative emotions (e.g., anger and depression) and improve emotional control compared to frequently used interventions (e.g., REBE interventions) (11, 24, 66, 73). While the therapeutic effect of MindLight on anxiety in children and adolescents is comparable to commercial VGs (37, 77) or CBT (76, 78), the extent of improvement of depression is found to be greater in adolescents who play SPARX than those who receive frequently used treatments (e.g., counseling) (58) and to be comparable to that of the CBT-based prevention approach (8).

**Table 2:** The summary of 21 studies that examined the therapeutic effects of DTx for children and adolescents.

**Study & DTx**	**Participants**	**Measures**	**Follow-up**	**Results**	**Limitations**
Anguera et al. (60) EndeavorRx	1. Children with SPD + IA (N_Experiment1_ = 20, N_female_ = 8, M_age_ = 9.7 ± 1.3; N_Experiment2_ = 20, N_finalsample_ = 17). 2. Age- and gender-matched children with SPD (N_Experiment1_ = 17, N_female_ = 8; M_age_ = 10.3 ± 1.5; N_Experiment2_ = 13, N_finalsample_ = 10). 3. Typically developing children (N_Experiment1_ = 25, N_female_ = 12, M_age_ = 10.5 ± 1.3; N_Experiment2_ = 24, N_finalsample_ = 22).	Behavioral measures - Perceptual discrimination paradigm - TOVA - EVO assessment (i.e., perceptual discrimination, visuomotor tracking and multitasking ability) - Parent report measure Vanderbilt ADHD Diagnostic Parent Rating Scale Neural assessment - EEG Additional measure for confounding variables - Basic response time task	9 months	Behavioral changes in cognitive control - Improved performance in the perceptual discrimination in children with SPD+IA and typically developing children - Improved performance in the TOVA in all groups - Improved reaction time in EVO assessments in all groups - No group difference in basic response time task Parent report of attention - Significantly reduced inattention behaviors in children with SPD+IA after the training and at 9-month follow-up Neural assessment - Significantly increased MFT power in children with SPD+IA - A correlation between the improvement of MFT power and decreased parental reports of inattentive behaviors	- The lack of power for the dissociation of attention-based improvements on behavioral measures - The lack of SPD placebo control group
Davis et al. (42) EndeavorRx	Children who were aged between 8–12 years and whose functioning was within the normal range of intellectual functioning	Safety and feasibility measures - Parental reports of adverse events - Compliance	No follow-up	Safety and feasibility - The completion of 84% of prescribed in-home sessions across all participants - Reports of 9 adverse events that were not related to EVO over all study phases	- The necessity of additional measures for the translation of cognitive improvement to everyday functioning - The plausible influence of parent expectations for the intervention treatment effects on the observed
	1. Children with ADHD (*N* = 40, N_female_ = 16; M_age_ = 10.3 ± 1.2) 1–2. ADHD high severity subgroup(*N* = 22, N_female_ = 8; M_age_ = 10.2 ± 1.2) 2. Age-matched typically developing children (*N* = 44, N_female_ = 19, M_age_ = 10.5 ± 1.4; N_finalsample_ = 40)	- Questionnaire on intervention appeal Measures for attention and other cognitive functions - TOVA - BRIEF–Parent Form CANTAB		-6.9 of enjoyment rating for EVO on a 1-10 scale Attention functioning - Significantly improved performance in API of the TOVA in children with ADHD and a greater intervention effect in ADHD high severity subgroup - Significant improvements on indicators of ADHD severity (i.e., Reaction Time Mean Standard Score and Reaction Time Variability Standard Score) only in the ADHD high severity subgroup Cognitive functioning - Improved spatial WM in children with ADHD and the improvement in rapid visual processing and delayed match to sample in typically developing children - Non-significant improvement of WM in children with ADHD and the significant improvement of WM and inhibition in the ADHD high severity subgroup - No changes in summary scores of BRIEF (i.e., metacognition, behavioral regulation, global executive composite) over time in all groups	improvement in parent reports - The limited generalizability of the findings to the entire general population of children with ADHD - Concern for Type 1 error for some results despite the Bonferroni correction for significant values
Yerys et al. (57) EndeavorRx	Children with ASD and ADHD (N_screened_ = 42, age range = 9–15 years;	Measures of attention and cognitive functions	No follow-up	Feasibility and acceptability	-The use of control treatment condition that completely differed from the experimental condition
	N_eligible_ = 19, N_female_ = 2, age range = 9–13 years, M_age_ = 11.25 ± 1.44) 1. Multi-tasking treatment group (*N* = 11) 2. Alternative educational treatment group (*N* = 8)	Primary outcome measure: TOVA - ADHD-RS-IV: Home - BRIEF, 2^nd^ edition - CANTAB - SSIS		-A high level of adherence to the treatment protocol in both groups and no withdrawal of children from the study - Positive reports for the benefits of the intervention in both children and adolescents Preliminary Efficacy - Non-significant but improved performance in the TOVA API in the multi-tasking treatment group and non-significant but overall worsening in the TOVA API in the alternative educational treatment group - Significant reductions in ADHD-RS-IV, BRIEF-2, SSIS problem behaviors in the multi-tasking treatment group but non-significant reductions in ADHD-RS-IV, BRIEF-2 and SSIS problem behavior scores in the alternative educational group - No significant gains in CANTAB's spatial WM and SSIS Social skills in both groups	-The plausible influence of parent perception of engagement on the parent ratings that were conducted after the treatment - The exclusion of a large percentage of children with ADHD at the screening stage - Some concerns of the extent to which TOVA measures cognitive interference
Kollins et al. (44) EndeavorRx	Children with ADHD (N_screened_ = 857, N_eligible_ = 348, age range: 8–12 years)	Primary outcome measure - The mean change in TOVA Secondary outcome measure	No follow-up	Feasibility - No parent reports of serious adverse events related to the intervention but some reports of frustration (3%) and headache (2%) in AKL-T01 group	-The exclusion of a substantial number of children with ADHD due to the criteria for attention deficit - The limited generalizability of findings to the broader populations of children with ADHD with comorbid condition or taking medication for ADHD
	1. AKL-T01 group (*N* = 180, N_female_ = 55, age = 9.7 ± 1.3; N_finalsample_ = 169) 2. Control group (*N* = 168, N_female_ = 45, age = 9.6 ± 1.3; N_finalsample_ = 160)	-IRS - ADHD-RS-IV - CGI-I - BRIEF		-No dropout in both groups due to adverse events Primary outcome - Significant improvement in TOVA API score in AKL-T01 group but not in control group - The greater improvement in the TOVA API in the AKL-T01 G compared to the control group with the movement of more children with ADHD into the normative ranges of attention functioning after the intervention Secondary outcomes - Significant improvement in all secondary measures but no group differences in secondary measures (i.e., IRS, ADHD-RS, ADHD-RS-I, ADHD-RS-H, BRIEF-Parent Inhibit and Working Memory and Metacognition) - Significantly higher parental-reported improvement of attention in the AKL-T01 G - Significant effects on ADHD-RS, ADHD-RS-I and CGI-I in children who discontinued stimulant medication for AKL-T01 intervention	-Concerns for the beneficial effects of the intervention on attention in different intervention periods or after the intervention period - No power calculations for secondary outcomes or post-hoc analyses - No collection of mechanistic data (e.g., EEG)
Gallen et al. (71) EndeavorRx	28 children with ADHD (N_finalsample_ = 25,	Primary measure	No follow-up	Neural assessment outcome	-No placebo control group for the comparison
	N_male_ = 20, M_age =_ 10.44 ± 1.23, age range: 8–12)	-Neural assessment: EEG recording during a perceptual discrimination task (i.e., go/no-go task) Behavioral measures - Perceptual discrimination task - Sustained attention task (i.e., visual continuous performance task) Parent-report measure - Vanderbilt ADHD diagnostic parent scale		-Increased MFT activities at the early and later stages of a go/no-go task but non-significant increases in MFT activities in the peak time of the task Behavioral assessment outcomes - Significantly improved reaction time in perceptual discrimination task after the intervention - No improvement in reaction time or reaction time variability in sustained attention task but the improvement in attention lapses - No relation of basic response time changes with intervention-related improvements in behavioral outcomes Parent-reports - Significant decreases in parent-reported ADHD inattention symptoms Relationship between neural and behavioral assessments - A significant relation of MFT-changes in early and post stages with the improvement of attention lapses in the sustained attention task	-Concerns for the persistency of the neural effects and the uncertainty in the relationship of the neural effects with aforementioned behavioral measures for attention - No correction for multiple comparisons in the reported association between neural and behavioral gains - The necessity of the replication of a lager sample of the heterogeneous ADHD population
Lim et al. (4) ATENTIVmynd	Children who were diagnosed to have	Neural measure	3 months with three once-monthly booster training sessions	Adherence	-The possibility of the exaggeration of the treatment effect due to uncontrolled open-label design of the study
	ADHD without the experience of stimulant medication treatment (N_enrolled_ = 25; N_eligible_ = 20, N_female_ = 4, age range: 6–12 years, M_age_ = 7.8 ± 1.4). - children with the combined subtype of ADHD (*N* = 14). - children with inattentive subtype of ADHD (*N* = 6).	- BASM Outcome measure - ADHD-RS completed by parents		- The completion of the study in 85% participants (N=17) Neural outcome - Non-significant but increasing trend in the mean BASM scores (i.e., the improvement of inattentive symptoms) in Week 20 (i.e., the post-boosters period) compared to in Week 0 (i.e., baseline) Behavioral outcomes Significant decreases in inattentive symptoms, hyperactive impulsive and combined symptoms immediately after the intervention - No significant further improvement in inattentive or hyperactive-impulsive symptoms of ADHD by receiving monthly booster trainings for 3 consecutive months after the intervention but sustained intervention effect at 24 weeks - Greater improvements in attention in children with more severe inattentive, hyperactive-impulsive and combined symptoms immediately after the intervention	
Qian et al. (72) ATENTIVmynd	Boys with ADHD (either combined or inattentive subtypes)	Neuropsychological measures	No follow-up	Neuropsychological outcomes	-A relatively small sample size after removing poor-quality data that resulted from the excessive motion
	1. Intervention group (*N* = 44, N_finalsample_ = 18, M_age_ = 9.00± 1.50) 2. Control group (*N* = 22, N_finalsample_ = 11, M_age_ = 9.45± 1.29)	-ADHD-RS - CBCL Neural measure - rs-fMRI and structural MRI		- Significantly greater reduction in inattention scores of the ADHD-RS in the intervention group compared to the control group - Non-significant but slightly greater reduction in CBCL internalizing problems in the intervention group than in the control group Neural outcome - A trend of increased functional connectivity within and between relevant networks (e.g., salience/ventral attention network, dorsal attention networks) only in control group over time - Significant reductions in nodal degree and clustering coefficient but significantly increased nodal closeness in salience/ventral attention network, executive control network and default mode network after the intervention - The correlation of changes in functional networks (i.e., less increased functional connectivity in the intra-salience/ventral attention network and the inter-network between salience/ventral and dorsal attention networks and reduced local functional processing in relevant brain regions) after the intervention with the	-The possibility of further correction of physiological noise in the fMRI dataset through the implementation of advanced fMRI preprocessing techniques ADHD subtypes that were not distinguished due to relatively small sample size - The necessity of further studies with a longer duration of the intervention and follow-ups
				improved inattention symptom and internalizing problems in children with ADHD	
McDermott et al. (20) ATENTIVmynd	46 children with ADHD (N_female_ = 14; age range = 8–12 years, M_age_ = 9.57 ± 1.34) 1. Intervention group (*N* = 21) 2. Waitlist group (*N* = 19) *Children who dropped during the training or wait period (*N* = 6)	Behavioral measures - ADHD-RS completed by clinicians - CGI-I - Quotient^®^ ADHD system Academic measures - PERMP - WJ-III	3 months	Behavioral outcomes - Significant decreases in both ADHD-RS mean scores and mean score of CGI in the intervention group - Worsening performances of the intervention group in Quotient^®^ ADHD system which was found to be not correlated with behavioral reports or training-related improvements - The maintenance of intervention-related improvements at the follow-up Academic outcomes - Significantly improved performance in the PERMP in the intervention group compared to waitlist group - A trend of improved performance in WJ-III understanding directions (i.e., the ability to control their impulses and follow directions) in the intervention group but no group differences in Reading fluency and Math fluency	-Potential expectation bias of clinicians and parents on the intervention effect due to non-blind group assignment - No comparison of the intervention against one evidence-based treatment due to various options of treatments, including no treatment, that were given to the control group - No comparison group at the follow-up - Limited generalizability of the findings due to relatively small sample size
García-Baos et al. (63) RECOGNeyes	28 Children and adolescents with ADHD (including 7 children with comorbid dyslexia and 2 children with a	Acceptability measure - A usability and enjoyability questionnaire	No follow-up	Acceptability outcome - Reports of appropriateness of difficulty level and enjoyment in the majority of children who were younger than 12 years	- Some group differences in the number and duration of fixations in the short word task at the baseline - The necessity for a longer period of intervention with a larger sample size
	learning disability; N_boy_ = 18, age range: 8–15 years) 1. RECOGNeyes group 2. Age-matched control group using mouse to play the game	Behavioral measures - frog task (i.e., probability of ADHD, severity of ADHD, hyperactivity index, impulsivity index) - word recognition task (i.e., dyslexia index, performance parameters)		Behavioral outcomes - Significantly decreased impulsivity score in the RECOGNeyes group after the intervention - Significantly decreased reaction time for attention, increased durations of fixations and decreased number of fixations in attention assessment tasks in the RECOGNeyes group after the intervention	
David et al. (11) REThink	Children and adolescents (*N* = 165, N_finalsample_ = 142, N_female_ = 91; age range: 10–16 years) 1. REThink group (*N* = 48; M_age_ = 13.0 ± 2.0) 2. REBE group (*N* = 48; M_age_ = 12.7 ± 1.9) 3. Waitlist group (*N* = 46; M_age_ = 12.9 ± 2.2)	Acceptability measure - TS-VAS Primary measures - SDQ—child version - The subscale of depressive moods from EATQ-R Secondary measures - ERICA - Other three subscales (i.e., attention, fear, inhibitory control) of EATQ-R - FD-CMS—girls and boys versions	No follow-up	Acceptability outcome - Significant difference in satisfaction between REThink and REBE group in the middle of the intervention but no-significant group difference in the satisfaction after the intervention Primary outcomes - Significantly decreased scores of SDQ emotional symptoms and EATQ-R depressive mood Secondary outcomes - Significantly increased ERICA awareness in the REThink group - Significantly increased scores of EATQ-R attention and ERICA control in the REThink group and the REBE group - Marginally significant group difference in SDQ peer-relationship problems	The possibility of the lack of significant results for REBE intervention due to the delivery of the intervention in schools - The necessity for the follow-up - Validation issues of some measures (e.g., FD-CMS)
David et al. (73) REThink	Children and adolescents (*N* = 165, N_finalsample_ = 142, N_female_ = 91; age range: 10–16 years) 1. REThink group (*N* = 48, N_female_ = 36; M_age_ = 13.0 ± 2.0) 2. REBE group (*N* = 48, N_female_ = 31; M_age_ = 12.7 ±1.9) 3. Waitlist group (*N* = 46, N_female_ = 22; M_age_ = 13.0 ± 2.2)	Primary measures - SDQ—child version - EATQ-R Measures for hypothesized mediating variables - CASI - CATS-N/P	No follow-up	- No change in CASI total score in the waitlist group and marginal, non-significant change in CASI total score in the REBE group but significantly decreased CASI total score in the REThink group -The significant association of changes in CASI total score with SDQ emotional symptoms and EATQ-R depressive symptoms in the REThink group -The prediction of group contrast (i.e., the REThink group or the waitlist group) for changes in CASI total scores, SDQ emotional symptoms and EATQ-R depressive mood -No difference in the intervention efficacy depending on the age or gender	- The usage of standard assessment points -The delivery of interventions in schools and children's previous exposure to such intervention programs
David et al. (24) REThink	Children and adolescents (*N* = 165, N_finalsample_ = 134; age range: 10–15 years) REThink G (*N* = 47, N_female_ = 34; M_age_ = 13.04 ± 2.07) REBE G (*N* = 42, N_female_ = 23; M_age_ = 12.81 ± 1.92) Waitlist G (*N* = 45, N_female_ = 21; M_age_ = 12.96 ± 2.17)	Report measures -‘concern and anxiety' subscale of PAD Neural measures - EEG data of frontal brain regions during the impromptu speech task	No follow-up	Report outcome - Significant decreases in state anxiety in all groups after the intervention Neural outcome - Increased frontal alpha asymmetry scores in the REThink group after the intervention - Significant negative relationship between frontal alpha asymmetry and state anxiety after the REThink intervention	- The limited generalizability of this findings based on a convenience sample - The dropout of participants that resulted from the inherent limitation of the used equipment - No validation of the anxiety subscale of the PAD in children and adolescent samples - The lack of control on the metacogntion for children and adolescents in the subjective measure
Ducharme et al. (66) Mightier	Children and adolescents who did not show the improvement in the anger control with treatment as usual (age range: 9–17 years) 1. Intervention group that had received anger control therapy with mightier (*N* = 20, N_finalsample_ = 18, N_female_ = 13; M_age_ = 13.7 ± 2.1) 2. Treatment as usual group that had received anger control therapy (*N* = 19, N_female_ = 9; M_age_ = 14.7 ± 2.4)	Acceptability measure - The therapeutic helpfulness questionnaire Behavioral measure - STAXI-CA Physiological measure - Heart Rate - The therapeutic helpfulness questionnaire	No follow-up	Acceptability outcome - A high level of satisfaction and helpfulness with the treatment in the intervention group Behavioral outcome - Significantly decreased scores of STAXI-CA subscales (i.e., state anger, trait anger and anger expression-out) and marginally decreased scores of anger expression-in subscale in the intervention group Physiological outcome - Significantly improved ability to maintain their heart rate in the intervention group	- The small sample size - The use of not a randomized control but a historic control group and the lack of cultural diversity of the experimental group - No inclusion of objective measures for anger or aggression
Ducharme et al. (74) Mightier	One 16-years-old girl who disclosed depression, suicidal ideation and thoughts of hurting family members	Acceptability measure - Revised session reaction scale Behavioral measure - STAXI-CA	No follow-up	Acceptability outcome - Positive feedback on the intervention experience (e.g., enjoyment, helpfulness) Behavioral/Report outcomes - Decreased scores of State Anger and Trait Anger after the intervention - Considerable improvement in the ability to use emotional regulation skills and to control heart rate after the intervention	-Engagement issues of patients with anger problems in the intervention - Difficulty to determine the mechanisms that led to the intervention-related improvement - Unknown generalizability of the improvement to the contexts outside the therapeutic settings - The necessity of randomized clinical trials - Plausible constraints (e.g., the necessity of managed care for more than five consecutive days) on the benefits of the intervention
Schoneveld et al. (37) MindLight	Children showing elevated anxiety symptoms (N_screened_ = 757, N_finalsample_ = 136, N_female_ = 73; age range: 8–13 years, M_age_ = 9.95 ± 1.33) 1. MindLight group (*N* = 9, N_dropout_ = 1) 2. Control game (i.e., “Max”) group (*N* = 67, N_dropout_ = 1)	Acceptability and feasibility measures - Game expectations - Game evaluations Behavioral measures - SCAS-C/P Additional measure - Average weekly time for game playing	3 months	Acceptability and feasibility outcomes - No group difference in game expectations at the baseline - The evaluation of MindLight as a significantly more anxiety-inducing game compared to Max and the evaluation of Max as a significantly more appealing game compared to MindLight Behavioral outcomes - Significantly lower mean scores on total anxiety symptoms in the MindLight group than in the control game group at the post-test but non-significant effect of game conditions on anxiety outcome measures - No group difference in anxiety symptoms at the follow-up; significant decreases in levels of anxiety symptoms in both groups over time	-The potential bias in report measurements - The possibility of the contamination between the groups due to the play context where both groups played in the same room - The exclusion of a passive control group - The lack of the understanding about reasons for the improvement in children's anxiety symptoms
Schoneveld et al. (76) MindLight	Children showing elevated anxiety symptoms (*N* = 174, N_female_ = 103; age range: 7–12 years, M_age_ = 9.97 ±1.16) 1. MindLight group (*N* = 86, N_finalsample_ = 72) 2. CBT group (*N* = 88, N_finalsample_ = 72)	Acceptability and feasibility measures - Program expectations before the group allocation - The evaluation of the program the participants received Behavioral measures - SCAS-C/P Additional measure - Average weekly time for game playing	3 months and 6 months	Acceptability and feasibility outcomes - The children's ratings of MindLight and the CBT program as equally appealing interventions across time points - The rating of the CBT program as more relevant to daily lives than MindLight Behavioral outcomes - Significant decreases in anxiety symptoms over time and slowed rate of the decrease over time - No sigfnicant group difference in anxiety symptoms after the intervention and at follow-ups	- The plausible influence of the information that was given to children and parents to equalize expectations across the conditions on the results - No available information about the mechanisms of the effectiveness of the interventions
Wijnhoven et al. (77) MindLight	Children and adolescents with ASD who showed at least subclinical level of anxiety symptoms (*N* = 109; age range: 8–16 years, M_age_ = 11.10 ± 2.07) 1. MindLight group (*N* = 53) 2. Control game (i.e., “Triple Town”) group (*N* = 56)	Acceptability measure - PETS Primary outcome measure - SCAS-C Secondary outcome measures - SCAS-P - ADIS-P	3 months	Primary outcome - No significant group differences in child-rated anxiety symptoms after the intervention Secondary outcomes - No significant group difference in parent-rated anxiety symptoms after the intervention but significantly decreased parent-rated anxiety symptoms in the MindLight group compared to the control game group at the follow-up - No group difference in remission rates	- Some concerns for drop-outs and late completion of assessments - The weak original model fits due to the sample size despite the correction - No available information for mechanisms that contributed to the improvement of anxiety symptoms - Some concerns for the reliability of SCAS-C in children with ASD - The interference of the research team in the content of treatments that children received in parallel with MindLight or the control game, and the plausible effects of the treatment contents on anxiety symptoms
					- The usage of ADIS-P as the measurement for the remission rates of anxiety disorders but not as an indicator for changes in anxiety severity
Wijnhoven et al. (78) MindLight	Children with ASD who showed at least subclinical level of anxiety symptoms (*N* = 8, N_female_ = 1; Age range: 8–12 years)	- Primary outcome measure - SCAS-C Secondary outcome measures - SCAS-P - ADIS-P - CSLK	3 months	Primary outcome - Clinically significantly decreased anxiety symptoms in five participants during the MindLight intervention Secondary outcomes - Remission of some phobias (e.g., social phobia) and/or generalized anxiety disorder in five children at the follow-up compared to the baseline - Significantly decreased avoidance in three children for more than one phases of the study - Significantly increased positive coping skills (e.g., direct problem solving) in three children, mixed changes in positive coping skills in two children and significantly decreased positive coping skills in one children	- The difficulty to find a significant additive effect of CBT due to the improvement of anxiety symptoms in some children during MindLight sessions - The plausible subjectivity in the visual analysis of data
Merry et al. (58) SPARX	Children and adolescents with mild to moderate depressive disorder (N_screened_ = 213, N_finalsample_ = 187; age range: 12–19 years)	Primary outcome measure	3 months	Acceptability and feasibility - Low dropout rates in both groups - A good level of adherence in SPARX group with the completion of at least four modules of SPARX in 86% participants	-The insufficient power of the study to detect the superiority of SPARX over treatment as usual
	1. SPARX group (*N* = 94, N_dropout_ = 9, N_female_ = 59; M_age_ = 15.55 ± 1.54) 2. Treatment as usual group (*N* = 93, N_dropout_ = 8, N_female_ = 64; M_age_ = 15.58 ± 1.66)	-CDRS-R Secondary outcome measures - Reynolds adolescent depression scale-second edition - mood and feelings questionnaire - pediatric quality of life enjoyment and satisfaction questionnaire - Spence children's anxiety scale - Kazdin hopelessness scale for children 1. CGI		- The report of 49 adverse events overall but mostly unrelated to the study - The report of helpfulness and satisfaction regarding the interventions in the majority of both groups Primary outcomes - Non-significant but greater reduction in mean scores of CDRS-R in the SPARX group compared to the treatment as usual group - Significantly higher remission rates on the primary outcome in SPARX group than in treatment as usual group Secondary outcomes - Significantly higher changes in mean scores of measures (i.e., Kazdin hopelessness scale, mood and feelings questionnaire and the Spence generalized subscale) in the SPARX group compared to the treatment as usual group but no group difference in the clinical global impression-improvement response rate - Maintained treatment effects in SPARX G at 3-month follow-up	- Logistic difficulty of adding more than one measure on the interview due to various issues (e.g., the necessity of extra time) - No exclusion of spontaneous improvements in both group due to the study design - The heterogeneity of the treatment as usual group
Fleming et al. (79) SPARX	Adolescents with depressive symptoms in alternative schooling	Primary outcome measure	10 weeks	Acceptability outcome	Small sample size - Short follow-up period
	programs (N_screened_ = 49,N_finalsample_ = 32; age range: 13–16 years, M_age_ = 14.9 ± 0.79) 1. SPARX group (*N* = 20) 2. Waitlist group (*N* = 12)	- CDRS-R Secondary outcome measures - Reynold Adolescents Depression Scale - Pediatric Quality of Life Enjoyment and Satisfaction Questionnaire - Spence Anxiety Scale - Kazdin Hopelessness Scale - Children's Nowicki-Strickland Internal-External Control Scale short form		-Report of six adverse events (i.e., increased depressive symptoms in four participants in the waitlist group and two self-harm incidents unrelated to the intervention) Primary outcome - Significantly greater decreases in scores of CDRS in the SPARX group than in waitlist group immediately after the intervention - Non-significant changes in outcomes between post-intervention and 10-week follow-up in the SPARX group Secondary outcomes - Significantly greater decreases in Reynold adolescents depression scale in the SPARX group compared to the waitlist group immediately after the intervention but non-significant changes in other secondary outcomes	- Post-intervention and follow-up assessments conducted by the researcher who was not blinded - No validation of used outcome measures in this specific group
Poppelaars et al. (8) SPARX	Girls with elevated depressive symptoms (*N* = 208; age range: 11–16 years, M_age_ = 13.35 ± 0.71) 1. OVK (i.e., a depression prevention program) group (*N* = 50) 2. SPARX group (*N* = 51)	Acceptability measure - Evaluation of the program at post-test Outcome measures - Reynolds adolescent Depression Scale - Item 9 (i.e., suicidal ideation) of CDI	3-, 6-, and 12-month	Acceptability outcome -Similar level of satisfaction in OVK and SPARX programs - The rating of OVK as a more attractive and useful intervention than SPARX Outcomes - Significant decreases in depressive symptoms over time from the screening to the follow-up in all groups	-The limited genralizability of the findings and the limited random allocation of participants into each group - Small sample size for subgroups of adolescents to be distinguished for the identification of a more effective program - The exclusion of male adolescents by focusing on female adolescents who were at higher risk for depression
	3. OVK and SPARX combined group (*N* = 56) 4. Monitoring control group (*N* = 51)			Similar rates of changes in depressive symptoms in all groups and similar level of depressive were at higher risk for depression symptoms in all groups at one-year follow-up - The prediction of greater depressive symptoms at the one-year follow-up from greater depressive symptoms at the baseline - No group difference in suicidal ideation	-Unknown mechanisms for the changes resulted from the intervention - Lack of understanding about the effectiveness of each aspect of games in the context of interventions - The deviation of depression measurements used in the current study from other studies
Lucassen et al. (69) SPARX	Children and adolescents (age range: 12–19 years) 1. Transgender adolescents (*N* = 294; Age 12–15 = 131, Age 16–19 = 76) 2. Male adolescents (*N* = 4,135; Age 12–15 = 1,927, Age 16–19 = 977) 3. Female adolescents (*N* = 9,060; Age 12–15 = 3,595, Age 16–19 = 2,373)	- Patient health questionnaire –modified for adolescents	No follow-up	- Low completion rates (i.e., the completion of Module 4 in less than 10% of participants and the completion of Module 7 in less than 4% of participants - Significantly improved scores of patient health questionnaire modified for adolescents in male and female adolescents after the intervention but no change in scores in transgender adolescents	- The low completion rates - The restricted single sex/gender item in the used self-report (i.e., the limited effectiveness of the measurement for transgender adolescents)

IA, inattention; TOVA, test of variables of attention; BRIEF, Behavior Rating Inventory of Executive Function; CANTAB, Cambridge Neuropsychological Test Automated Battery; API, Attention Performance Index; ADHD-RS, ADHD rating scale; SSIS, social skills improvement system; IRS, impairment rating scale; CGI-I, Clinical Global Impressions-improvement; BASM, BCI ADHD severity Measure; CBCL, Child Behavior Checklist; PERMP, Permanent Product Measure of Performance; WJ-III, Woodcock-Johnson Third Edition; SDQ, Strengths and Difficulties Questionnaire; ERICA, Emotion-Regulation Index for Children and Adolescents; EATQ-R, Early Adolescent Temperament Questionnaire97Revised; FD-CMS, Functional and Dysfunctional Child Mood Scales; TS-VAS, Treatment Satisfaction Visual Analogue Scales; CASI, Child and Adolescent Scale of Irrationality; CATS-N/P, Children's Automatic Thoughts Scale-Negative/Positive; PAD, Profile of Affective Disorder; SCAS-C/P, Spence Children's Anxiety Scale - child & parent versions; ADIS-P, Anxiety Disorders Interview Schedule for DSM-IV, Parent version; PETS, Parent Expectancies for Therapy Scale; CSLK, Coping Strategies Checklist for Children; STAXI-CA, State Anger, Trait Anger, Anger Expression-Out, Anger Expression-In & Anger Control; CDRS-R, Children's depression rating scale-revised; OVK, Op Volle Kracht.

## The sustainability of therapeutic effects of DTx

Therapeutic effects of DTx were found to be sustained at follow-ups. The treatment effects of ATENTIVmynd, MindLight and SPARX on targeted mental health problems in children and adolescents were maintained for 3 months after the interventions (4, 20, 37, 58, 76, 77). The effectiveness of SPARX on depression was also maintained in children who were excluded from mainstream education at the 10-weeks follow-up (79). Moreover, the treatment effects of some DTx were maintained for longer durations. Larger decreases in anxiety symptoms that were reported by children who played MindLight were maintained at the 6-months follow-up and the sustained improvement in anxiety symptoms was reported by both children and their parents (76). Unlike the sustained improvement of anxiety symptoms in both children who played MindLight and those who played the control game, “Max”, at the follow-up (37), not parents of children, who played the control game (i.e., “Triple Town”), but parents of children, who played MindLight, reported the sustained decreases in their anxiety symptoms at the follow-up (77). In case of EndeavorRx, its therapeutic effects on attention function in children with SPD and inattentive-ADHD were sustained for 9 months after the intervention (60). Furthermore, receiving three booster training sessions of ATENTIVmynd during the 3-months follow-up did not show the further improvement of attention function at the follow-up (4). That is, the therapeutic effect of ATENTIVmynd on attention function was maintained without the additional training sessions (4). However, the extent of improvement of anxiety symptoms was lesser in children who played MindLight and reported the highest weekly engagement time in gaming (76). Taken together, although the sustainability of RECOGNeyes, REThink, and Mighter was not examined, EndeavorRx, ATENTIVmynd, MindLight, and SPARX were found to show sustained therapeutic effects on targeted mental health problems (i.e., attention function, anxiety and depressive symptoms) for at least 3 months outside the intervention period.

## The transfer of cognitive functions in DTx

When the extent to which cognitive functions were improved in game-based DTx was investigated, they showed the near transfer of cognitive functions. Both children with ADHD and typically developing children showed improvement in spatial working memory (WM) after playing EndeavorRx (42). However, the significant improvement in WM and inhibition after EndeavorRx intervention was found not in typically developing children but in children with ADHD (42). Despite the trend of improvement in WM in children with ADHD, children with a more severe level of ADHD showed the significant improvement in WM and inhibition (42). Children, who played REThink, and those, who received REBE intervention, also showed the significant improvement in focused attention (11). However, the extent of improvement of cognitive functions was limited to the characteristics of game-based DTx. Children, who played ATENTIVmynd, showed the improvement in focused attention by staying on tasks, completing more questions in the time limit and correctly answering more questions compared to those in the control group but did not show improvements in reading and math fluency after playing ATENTIVmynd (20). That is, DTx that targeted attention deficit or aimed to prevent emotional problems through the development of emotion regulation showed the improvement in cognitive functions that were related to the targeted mental health problems.

## Discussion

This study aimed to review whether there are therapeutic effects of game-based DTx that are developed to target mental health problems in children and adolescents by delivering treatment or prevention interventions through VGs. It also reviewed whether the therapeutic effects of game-based DTx are sustainable and/or show the transfer of cognitive functions. Based on the review of literatures for the seven game-based DTx, it is found that using VGs as a delivery tool for treatment or prevention interventions in children and adolescents have the potential for therapeutic effects on targeted mental health problems (i.e., attention deficit, anxiety symptoms, emotion regulation and depression). The therapeutic effects of game-based DTx on mental health problems for this age group are at least comparable to that of frequently used treatment interventions (e.g., CBT) or are greater than some of the treatment interventions (e.g., REBE intervention). Moreover, game-based DTx show the persistent therapeutic effect of mental health problems in children and adolescents in at least short term (e.g., 3 months) and the near transfer of cognitive functions.

### Acceptability and feasibility

Implementing VGs in DTx that aim to treat or prevent mental health problems in children and adolescents appears to be acceptable and feasible. Among seven game-based DTx, EndeavorRx (42, 44, 57), ATRNTImynd (4), Mightier (66) and SPARX (58, 79) show a higher level of adherence to the interventions without the reports of serious adverse events that are related to the DTx intervention. The acceptability and feasibility of EndeavorRx are found in children with ADHD (42), children with ADHD and ASD (57) and children with SPD and inattentive symptoms (60). Mild adverse events (i.e., the frustration in 3% of children and the headache in 2% of the children) that were reported in EndeavorRx also do not result in the dropout of the study (44). In case of SPARX, although the lower adherence level of SPARX in the sample including transgender adolescents is found (69), the study that examined the feasibility and acceptability of SPARX in adolescents in inpatient settings suggests that SPARX is a feasible intervention for adolescent patients with a greater severity of mental health problems (70). Moreover, the positive evaluation for EndeavorRx, Recogneyes, REThink, and SPARX (e.g., the enjoyment, satisfaction and helpfulness) is reported (11, 45, 57, 58, 63, 66, 74). In case of RECOGNeyes, age influences the evaluation of the intervention (63). While children, who were younger than 12 years, reported that RECOGNeyes were enjoyable and challenging without difficulty, those, who were older than 12 years, reported that RECOGNeyes were not interesting to play (63). Furthermore, unlike the suggested potential of VGs as an attractive and immersive tool to deliver clinical interventions in children and adolescents (33, 34), MindLight and SPARX are found to be rated as interventions that are less relevant with daily lives and less useful than CBT or CBT-based interventions despite the comparable appealing or satisfaction level (8, 76). These results suggest the necessity of further studies to examine factors that could influence the acceptability and feasibility of game-based DTx (e.g., age). Taken together, as EndeavorRx is the firstly approved DTx that delivers the treatment for children and adolescents through VGs (56), more studies for the acceptability and feasibility of game-based DTx should be conducted.

### Immediate therapeutic effects

Despite the difference in the focus of DTx on the way to implement VGs, such as the focus on structural characteristics such as storylines [e.g., (37, 42)] and/or the controller to play VGs [e.g., (63, 66)], identified seven game-based DTx are found to be effective in treating or preventing targeted mental health problems in children and adolescents as standalone DTx [i.e., DTx that could treat targeted disorders independently (29, 80)]. It was because not only behavioral improvement in targeted mental health symptoms but also the alterations in symptom-relevant brain regions are found. Consistent with the potential of digital interventions for the cognitive rehabilitation (32), EndeavorRx (60, 71), ATENTIVmynd (72), and REThink (24) show alterations in brain regions that are associated with the behavioral improvement. However, in case of MindLight, although the extent of improvement of anxiety symptoms through MindLight intervention does not significantly differ from the improvement of anxiety through control commercial games (37, 77), MindLight appears to be an effective intervention to improve anxiety symptoms in children by offering a more anxiety-inducing environment where children could learn and practice emotion regulation strategies in response to triggered anxiety (37). The burdens of time investment, one of the reasons for dropping out of CBT program, are also not reported in MindLight (76). Moreover, the therapeutic or preventive effects of game-based DTx on targeted mental health problems are greater in children who report more severe levels of mental health problems than in those with less severe mental health problems (4, 42, 58, 60, 77). That is, children and adolescents, who are more likely to fail in receiving frequently used treatments or to report lower remission rates [e.g., children with SPD (60) and children with ASD in addition to ADHD (57)], show the improvement of targeted mental health problems after playing game-based DTx. These results suggest that DTx offer more personalized intervention environments where children and adolescents could experience the optimal level of challenge for their mental health problems (36, 37), resulting in the successful intervention results for those who show limited therapeutic effects through frequently used treatments (37, 41). Furthermore, the therapeutic effects of EndeavorRx and Mightier are found in children without attention deficits (42, 60) and those whose primary mental health problem is not the difficulty to control anger (66). Taken together, game-based DTx are suggested to show therapeutic or preventive effects on mental health problems by overcoming limitations of frequently used interventions. The DTx are also suggested to have the potential to not only be applied to children with mental health conditions that are relevant with targeted mental health problems but also improve cognitive functions in the general population of children and adolescents.

Although delivering treatment or prevention interventions for targeted mental health problems through VGs in DTx enables more personalized interventions for children and adolescents, personal factors (e.g., gender) seem to have the potential influence on the therapeutic effects of DTx. In SPARX, the extent to which hopelessness decreases and the quality of life improves is different between different groups of children and adolescents. Unlike the improvement in these aspects in children with mild to moderate level of depression (58), adolescents, who were excluded from mainstream education, did not report significant reduction in their hopelessness and improvement in life quality after playing SPARX (79). Moreover, unlike male and female adolescents, transgender adolescents also did not show the improvement in depressive symptoms through SPARX intervention (69). The potential influence of personal factors on the therapeutic effects of DTx could hinder the objective understanding for the effectiveness of game-based DTx in targeted mental health problems or could provide useful information for designing game-based DTx in the future. Thus, further studies should be conducted.

### The sustainability of therapeutic effects

Consistent with the potential that the implementation of VGs have for the generalization of learned skills (37), the improvement in targeted mental health problems (i.e., attention deficit, anxiety and depression) in children and adolescents are found to be sustained for more than 10 weeks after the interventions (4, 20, 37, 58, 60, 76, 77, 79). In case of MindLight, children showed greater improvement of anxiety symptoms than those, who received CBT, not at the 3-month follow-up but at the 6-month follow-up (76). That is, game-based DTx appear to have to potential to overcome the limited sustainable therapeutic effects of frequently used treatments outside the intervention period. The sustained therapeutic effects of DTx on mental health problems suggest the generalization of acquired knowledge or skills into daily lives (41) in that not only children but also their parents or clinicians report the improvement of targeted mental health problems (4, 37, 58, 76, 77). Moreover, as receiving additional training sessions of ATENTIVmynd outside the intervention period did not result in further improvement of attention function in children (4), offering the appropriate level of game-based DTx interventions is found to be sufficient to persistently improve targeted mental health problems in children and adolescents. However, the lesser extent of the sustained improvement in anxiety symptoms is found in children who played MindLight and reported the highest amount of gaming time per week (76). As the commercial game with structural characteristics that are similar to MindLight (e.g., controlling the avatar and requiring the overcoming of fear) show the sustained improvement of anxiety symptom at the follow-up (37), it seems that some structural characteristics of commercial VGs could help the improvement of anxiety symptoms or interfere with the sustainability of therapeutic effects of game-based DTx. In order to understand the interference between commercial game playing and game-based DTx, further studies examining the influence of structural characteristics of VGs on therapeutic effects of DTx should be conducted. Furthermore, gaming behavior is identified as one of factors that could influence the maintenance of therapeutic effects of DTx. The change and maintenance of anxiety symptoms through MindLight is found to be predicted by gaming behavior of children (68). While avoidant/safety behaviors predicted increased anxiety symptoms 3 months after the training, engaged gaming behaviors predicted the reduction in anxiety (68). That is, for the better understanding about the sustainability of game-based DTx, further studies should be conducted by investigating the types of engagement behavior and other plausible factors that could influence the sustainability of DTx.

### Cognitive improvements

Along with alterations in brain activations related to targeted mental health problems of EndeavorRx, ATENTIVmynd and REThink [e.g., inhibited activation in right frontal brain regions (24), increased MFT activity power (60), and reorganized functional networks (72)], cognitive functions that are relevant with targeted mental health problems (e.g., spatial WM, inhibition and focused attention) are found to show the improvement in children after playing EndeavorRx, ATENTIVmynd, or REThink (11, 20, 42). However, despite the relevance of the focused attention with academic performance, playing ATENTIVmynd does not result in the improvement in academic performance (e.g., reading) (20). That is, consistent with the near transfer of cognitive improvements that was found in VG playing (54), game-based DTx show the near transfer effects for the cognitive improvement. Moreover, although the improvement in spatial WM and inhibition were found in children with and without attention deficits after playing EndeavorRx, those, who reported more severe levels of attention deficit, showed the significant improvement in related cognitive functions along with the greater immediate therapeutic effects of EndeavorRx (42). That is, game-based DTx seem to booster therapeutic effects by improving not only targeted mental health problems but also relevant cognitive functions in children who have more difficulties to learn targeted skills in frequently used treatments. Further studies should be conducted to examine whether the extent of therapeutic effects of DTx in children with more severe mental health problems is modulated by the improvement of relevant cognitive functions.

## Limitations

Although both immediate and sustainable therapeutic effects of game-based DTx on targeted mental health problems in children and adolescents are found, four limitations are identified in this review. The first limitation is that the therapeutic effects of DTx are based on relatively small sample size. While more than 100 participants were included in some studies [e.g., (24, 37, 44, 58)], other studies in this review examined the therapeutic effects of DTx based on relatively small sample size. For example, the effectiveness of Mightier in the improvement of emotion regulation in children and adolescents was based on the study included 37 children (66) and one case study (74). As the results of the therapeutic effects of DTx based on small sample size showed the limited generalization of the findings (38), further studies with larger sample sizes should be conducted.

The second limitation is that the motivation of game playing and structural characteristics of VGs are not sufficiently considered. In order to ensure the therapeutic effects of MindLight on anxiety symptoms in children by controlling attention, motivation, behavioral activation and expectations (37, 81), commercial games were used as active control conditions (37, 77). However, game playing itself appears to provide children with the environment where they could train their anxiety reduction-related skills (e.g., resilience and self-efficacy) (35) and it is found that children, who report clinical level of mental health problems, are more likely to play games for the reduction of stress (82). As the motivation of game playing makes it difficult to understand the therapeutic effects of MindLight on anxiety, including additional control groups should be considered in future studies. Moreover, there are inconsistencies in the sustainability of improved anxiety between studies. While children, who played the commercial game, showed sustained improvement of anxiety at the follow-up like those who played MindLight (37), playing commercial games for longer time was found to decrease the extent of sustainability of therapeutic effects of MindLight (76). Thus, further studies that would explore the structural characteristics of VGs that could influence the immediate and/or sustained therapeutic effects of game-based DTx should be conducted.

The third limitation is that self-report measurements that ask children to explicitly indicate their suggestive states (83) are frequently used to examine therapeutic effects of game-based DTx in most studies. The explicit indication of subjective states could be challenging for them in that metacognitive insights are required (83). Although reports are also completed by their parents or clinicians in some studies [e.g., (58, 77)], physiological measurements (e.g., EEG) that are less sensitive to cognitive factors that could bias self-reports could offer more objective information (24) especially in studies where the blinding of conditions is limited. Among included research articles, only four studies [e.g., (24, 60)] are found to include the physiological measurements. As structural and functional alterations in brain regions were influenced by various experiences (e.g., physical activity and cognitive training) (84), investigating the alterations in brain regions after playing game-based DTx would provide valuable and objective information about their effectiveness in addition to behavioral evidence. That is, further studies that include physiological measurements should be conducted.

The last limitation is that the number of studies that examine the extent to which DTx are sustainable in short-term and long-term or show the transfer of cognitive functions is limited. Based on the short-term and long-term follow-ups for the investigation of the sustainability of game-based DTx, they are found to overcome the limited sustainability of frequently used treatments. However, most studies focus on the short-term maintenance of treatment effectiveness and the therapeutic or preventive effects of some DTx are not followed up. As the sustainability of the effectiveness of DTx outside the intervention period is one of important concerns that are reported in mental health interventions (43, 44), more studies that would follow up the therapeutic effects of DTx in long-terms should be conducted. Moreover, greater therapeutic effects of game-based DTx in children with more severe level of mental health problems and the significant improvement in cognitive functions that are related to targeted mental health problems (42) suggest the potential of cognitive remediation through game-based DTx that could result in greater therapeutic effects. Despite the potential of game-based DTx in cognitive remediation (32), only three studies that were included in this review [e.g., (46)] are found to examine the transfer of cognitive functions in game-based DTx. In order to ensure that game-based DTx show sustainable therapeutic effects and have additional effects on VG-related cognitive improvement, studies that would follow up the sustainability of therapeutic effects of DT in longer terms and that would investigate the extent of cognitive improvement after playing game-based DTx should be conducted in the future.

## Conclusion

Game-based DTx that aims to treat or prevent targeted mental health problems in children and adolescents are found to be acceptable and feasible. They are also found to show therapeutic or preventive effects on attention function, emotional regulation, anxiety symptom and depression. The therapeutic effects of game-based DTx are sustained in at least short term outside the intervention period. Cognitive functions that are related to the targeted mental health problems also show the improvement. However, there are factors that have the potential to influence the therapeutic effects of DTx or the sustainability of therapeutic effects. Moreover, there are limitations that should be considered in understanding the evidence for the therapeutic effects of DTx. In order to investigate the therapeutic effects of game-based DTx in children and adolescents more objectively, studies that consider the identified factors and limitations should be conducted in the future.

## Data availability statement

The original contributions presented in the study are included in the article/supplementary material, further inquiries can be directed to the corresponding author.

## Author contributions

EC, EY, and MP contributed to data collection, literature review, and the writing of the manuscript. EC was a major contributor in writing the manuscript. MP contributed to the planning, analysis, and supervision of the study. All authors have read and approved the final manuscript.

## Funding

This work was supported by the Technology Innovation Program (or Industrial Strategic Technology Development Program) (PC20ONDI0074, AI Driven Global PHR Pediatric Developmental Disability Management/Treatment Platform) funded by the Ministry of Trade, Industry and Energy (MOTIE, Korea) and by National Research Foundation of Korea (NRF) grant funded by the Korean Government (NRF- 2020R1F1A1070581). The funders did not have any role in study design, literature review, decision to publish, and preparation of the manuscript.

## Conflict of interest

The authors declare that the research was conducted in the absence of any commercial or financial relationships that could be construed as a potential conflict of interest.

## Publisher's note

All claims expressed in this article are solely those of the authors and do not necessarily represent those of their affiliated organizations, or those of the publisher, the editors and the reviewers. Any product that may be evaluated in this article, or claim that may be made by its manufacturer, is not guaranteed or endorsed by the publisher.
